# Transcriptomics and epigenetic data integration learning module on Google Cloud

**DOI:** 10.1093/bib/bbae352

**Published:** 2024-08-05

**Authors:** Nathan A Ruprecht, Joshua D Kennedy, Benu Bansal, Sonalika Singhal, Donald Sens, Angela Maggio, Valena Doe, Dale Hawkins, Ross Campbel, Kyle O’Connell, Jappreet Singh Gill, Kalli Schaefer, Sandeep K Singhal

**Affiliations:** Department of Biomedical Engineering, University of North Dakota, 501 N. Columbia Road Stop 8380, Grand Forks, ND 58202, United States; Department of Biomedical Engineering, University of North Dakota, 501 N. Columbia Road Stop 8380, Grand Forks, ND 58202, United States; Department of Chemistry and Physics, Drury University, 900 N. Benton Avenue, Springfield, MO 65802, United States; Department of Biomedical Engineering, University of North Dakota, 501 N. Columbia Road Stop 8380, Grand Forks, ND 58202, United States; Department of Pathology, University of North Dakota, 1301 N. Columbia Road Stop 9037, Grand Forks, ND 58202, United States; Department of Pathology, University of North Dakota, 1301 N. Columbia Road Stop 9037, Grand Forks, ND 58202, United States; Deloitte, Health Data and AI, Deloitte Consulting LLP, 1919 N. Lynn Street, Suite 1500, Arlington, VA 22209, United States; Google, Google Cloud, 1900 Reston Metro Plaza, Reston, VA 20190, United States; Google, Google Cloud, 1900 Reston Metro Plaza, Reston, VA 20190, United States; NIH Center for Information Technology (CIT), 6555 Rock Spring Drive, Bethesda, MD 20892, United States; NIH Center for Information Technology (CIT), 6555 Rock Spring Drive, Bethesda, MD 20892, United States; Department of Biomedical Engineering, University of North Dakota, 501 N. Columbia Road Stop 8380, Grand Forks, ND 58202, United States; Department of Biomedical Engineering, University of North Dakota, 501 N. Columbia Road Stop 8380, Grand Forks, ND 58202, United States; Department of Biomedical Engineering, University of North Dakota, 501 N. Columbia Road Stop 8380, Grand Forks, ND 58202, United States; Department of Pathology, University of North Dakota, 1301 N. Columbia Road Stop 9037, Grand Forks, ND 58202, United States

**Keywords:** transcriptomics, epigenomics, Google Cloud computing, multi-omics integration, DNA methylation, R Bioconductor

## Abstract

Multi-omics (genomics, transcriptomics, epigenomics, proteomics, metabolomics, etc.) research approaches are vital for understanding the hierarchical complexity of human biology and have proven to be extremely valuable in cancer research and precision medicine. Emerging scientific advances in recent years have made high-throughput genome-wide sequencing a central focus in molecular research by allowing for the collective analysis of various kinds of molecular biological data from different types of specimens in a single tissue or even at the level of a single cell. Additionally, with the help of improved computational resources and data mining, researchers are able to integrate data from different multi-omics regimes to identify new prognostic, diagnostic, or predictive biomarkers, uncover novel therapeutic targets, and develop more personalized treatment protocols for patients. For the research community to parse the scientifically and clinically meaningful information out of all the biological data being generated each day more efficiently with less wasted resources, being familiar with and comfortable using advanced analytical tools, such as Google Cloud Platform becomes imperative. This project is an interdisciplinary, cross-organizational effort to provide a guided learning module for integrating transcriptomics and epigenetics data analysis protocols into a comprehensive analysis pipeline for users to implement in their own work, utilizing the cloud computing infrastructure on Google Cloud. The learning module consists of three submodules that guide the user through tutorial examples that illustrate the analysis of RNA-sequence and Reduced-Representation Bisulfite Sequencing data. The examples are in the form of breast cancer case studies, and the data sets were procured from the public repository Gene Expression Omnibus. The first submodule is devoted to transcriptomics analysis with the RNA sequencing data, the second submodule focuses on epigenetics analysis using the DNA methylation data, and the third submodule integrates the two methods for a deeper biological understanding. The modules begin with data collection and preprocessing, with further downstream analysis performed in a Vertex AI Jupyter notebook instance with an R kernel. Analysis results are returned to Google Cloud buckets for storage and visualization, removing the computational strain from local resources. The final product is a start-to-finish tutorial for the researchers with limited experience in multi-omics to integrate transcriptomics and epigenetics data analysis into a comprehensive pipeline to perform their own biological research.

This manuscript describes the development of a resource module that is part of a learning platform named ``NIGMS Sandbox for Cloud-based Learning'' https://github.com/NIGMS/NIGMS-Sandbox. The overall genesis of the Sandbox is described in the editorial NIGMS Sandbox [16] at the beginning of this Supplement. This module delivers learning materials on the analysis of bulk and single-cell ATAC-seq data in an interactive format that uses appropriate cloud resources for data access and analyses.

**Highlights:**

- DNA methylation and gene expression are essential for unraveling the biological mechanisms underlying development, disease, and cellular function.
- Module will bridge a critical need in development tools for the next generation of bioinformaticians using cloud computing with modern multi-omics techniques.
- The three submodules (transcriptomics, epigenomics, and integration) provide deeper insights into the complexity of genetics and epigenetic regulation.

## Introduction

### Biological background

To effectively study the complex mechanisms of human health and disease requires the analysis and interpretation of molecular activity across as broad a biological hierarchy as possible, including the genomic environment, transcriptomic activity, protein expression, epigenetic modification, metabolite classification and concentration, environmental exposure, and microorganism interactions. Following the introduction of high-throughput sequencing technology, biological data of this type has become crucial for molecular, clinical, and translational bioinformatics research. The enormous amount of biologically meaningful quantifiable data generated at these hierarchical levels, collectively known as ‘multi-omics’ data [1], is rapidly changing how researchers analyze disease processes and is allowing for the creation of novel integrated systems-level approaches to understanding the complicated biological interactions in disease states, such as ovarian cancer [2], heart failure [3], lymphoma [4], Crohn’s disease [5], bladder cancer [6, 7], malaria [8], metabolic disorders [9], breast cancer [10–12], and countless more.

Combining data analysis across the different multi-omics regimes is becoming increasingly important for building a broader understanding of disease development in medical research and has resulted in the identification of novel biomarkers and molecular disease subtypes, the discovery of new targets for therapy, and the development of more targeted and personalized patient-specific treatments [13–16]. This work focuses on establishing a training protocol for integrating transcriptomic and epigenetic data utilizing the cloud computing infrastructure on Google Cloud, with an emphasis on practical application geared towards biological researchers with some background in genomics. Though genomic analysis is certainly indispensable and has historically been the focus of bioinformatics research, epigenetic information, which mounting evidence suggests plays an important role in human disease and cancer, corroborated with studies demonstrating epigenetic regulatory heterogeneity within and around the tumor microenvironment [17–19], is rarely quantified or measured at this level [20]. However, multi-omics analysis methods, such as the transcriptomics and epigenetic data integration and analysis pipeline, we will present here, are steadily gaining significance in understanding complex disease pathologies, such as cancer, from a cellular and molecular perspective. Transcriptomics analysis has already been established in the literature as a valuable tool for understanding cancer mechanisms and identifying prognostic, diagnostic, and predictive biomarkers [21], and since the heritable elements of disease can largely be explained through the interaction of genetic and epigenetic variation, to understand the relationship between the two regimes and how each can help predict and understand disease mechanisms is of the utmost importance to the earnest investigator.

In general, multi-omics research generates a vast amount of data, so it is vital that the user become acquainted with efficient computational platforms to manage and analyze this data. Cloud computing services like Google Cloud (GC) offer scalable and cost-effective solutions for data storage, analysis, and collaboration [22]. Our team has designed a cloud-based teaching module on GC to show investigators who possess limited familiarity with multi-omics approaches how to incorporate transcriptomics and epigenetics data analysis and integration protocols into a comprehensive analysis pipeline for their own work. The objective of the training module is not only to illustrate how transcriptomics and epigenetic data are combined but also why this is significant in biological research. The training module is composed of three sequential sub-modules that together form a task-oriented multi-omics tutorial for the user. The content covered in the tutorial is presented in the form of breast cancer case studies, and includes data retrieval, data processing, and downstream analysis. The scalability and flexibility of GC’s computational resources (and integrated tools for collaboration and storing and visualizing data) enable the analysis and processing of information by investigators to be performed expediently and at reasonable expense [23], making GC a robust bioinformatics research platform.

### Module overview

The instructional analysis pipeline consists of three submodules: RNA-seq (the transcriptomics module), RRBS (reduced-representation bisulfite sequencing, the epigenomics module), and the integration module. The first two submodules (RNA-seq and RRBS) are centered around preprocessing and differential analysis of transcriptomic and epigenomic data, while the third integration submodule focuses on combining the two multi-omics data types. The modules were created using GC and specifically designed to be used in conjunction with a cloud computing platform. The training protocols make extensive use of web-based computational environments capable of creating notebook documents, known as Jupyter notebooks. Note that the Jupyter notebooks utilized within the training module are compatible with other cloud platforms, such as Amazon AWS or Microsoft Azure, with only slight differences in how the data is stored and handled. Additionally, the tutorial process requires creating a virtual machine instance, which is simply an emulated computer system hosted on the Google infrastructure. Note that the procedure used to initiate the virtual machine will be slightly different if a cloud computing platform other than GC is used.

As mentioned, high-throughput sequencing technology has emphasized the importance of transcriptomics in revealing the complicated molecular landscapes, altered functions, and biochemical pathways associated with various biological systems [24, 25]. Utilizing multi-omics methodologies has enabled researchers to integrate transcriptomics information with data sets from different omics fields, aiding in understanding the molecular mechanisms, processes, and pathways discriminating health and disease [26], and assisting in uncovering novel molecular subtypes, finding biomarkers for prognosis, and informing investigations into targets of therapeutics and potential personalized treatment plans for patients [27]. There are many accessible resources and tools at hand for multi-omics analysis, from training modules demonstrating bioinformatics workflows to large databases, such as the Gene Expression Omnibus (GEO), for retrieving and sharing gene expression data, to cloud computing platforms, such as Google Cloud Storage, for efficiently managing and storing large amounts of unstructured data [28]. Furthermore, bioinformatics research has grown increasingly reliant on cloud computing, with an increasing demand for training a new iteration of bioinformatics investigators in these computational methods in order to fully exploit the potential for the scalable management of large biological data sets at reasonable expense [29].

This module employs RNA sequencing data within the presented training analysis pipeline. While many transcriptome sequencing applications exist, RNA-seq has been shown to most effectively, reliably, and flexibly ascertain gene expression and transcription activation at the genome level [30]. The differential expression accuracy, high-throughput capacity, and high resolution made possible with RNA-seq analysis have yielded noteworthy results and have been shown to be a critical tool in transcriptomics research over the last 10 years [31], which is especially important considering that fully understanding the biological differences underlying healthy and diseased states is dependent upon knowing how genes in a particular system are differentially expressed [32, 33]. This information can help investigators select gene expression targets for future research, discover new biomarkers, characterize the cellular tumor microenvironment, and identify the molecular pathways that cause phenotypic variability [34, 35].

The epigenomic data used in the training analysis pipeline presented here consists of DNA methylation profiles produced from RRBS. As we have discussed, the research community is becoming more and more interested in identifying the significance of epigenetic changes, such as DNA methylation, histone modification, and non-coding RNA (the three main epigenetic marks) [36], and their vital influence on complicated biological processes in the human cell, including how they regulate gene-expression to turn genes on/off and modify cell function, contributing to disease development [37]. Toward this end, increasing numbers of DNA methylation sequencing datasets from the human genome are being produced using various array and sequencing-based platforms, from methylated DNA precipitation to whole genome bisulfite sequencing [38], and many of those datasets are freely accessible for secondary analyses. The incorporation of information from the epigenome with omics data from additional fields using computational approaches is essential for a complete investigation of the molecular interactions that underpin complex biological pathologies [39], and demonstrating a comprehensive pipeline of analysis for this integration that the user can apply in their personal research is one of the main motivations for this work. It is essential to properly train the next iteration of epigenomics investigators in the computational and analytical methods necessary to efficiently manage the sizable amount of multi-dimensional data being created within the field and to keep up with scientific demand [40].

While integrative analysis of multi-omics datasets has proven to be extremely valuable to more thoroughly understanding complete biological systems, especially in cancer and precision medicine [41], obtaining multimodal data from the same samples is often difficult and expensive. Even with recent computational advancements, integrating multiple platforms of different omics data remains a formidable computational challenge. In order to explore the potential regulatory relationships between epigenetic modification, gene expression, and gene function, our module presents potential bioinformatics researchers with a comprehensive pipeline for a computational analysis workflow that can be used to explore the regulation mechanisms of genes that are both differentially methylated and exhibit differential expression in different phenotypic conditions via training on representative multi-omics data sets measured on the same set of biological samples. To explain the importance of our methodology, we would like to highlight our previously published research, wherein we studied the paired gene expression of primary breast tumors and DNA methylation data to identify a new sub-classification of breast cancer [42] described in the data discussion section of this manuscript.

## Method and implementation

### Technical requirements

**Figure 1:**
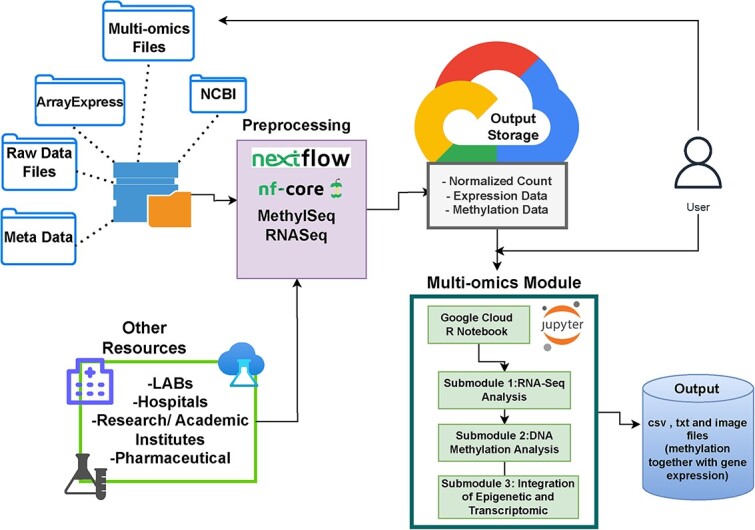
The figure illustrates the Google Cloud architecture used in the creation of the module, as seen from the perspectives of both the developer and the user. The figure demonstrates the essential elements used in the creation of modules, such as Google Storage Buckets, Nextflow, and R Jupyter Notebook. It also elucidates that users have the option to either commence from the beginning or utilize pre-existing modules to acquire knowledge about the integration of epigenetic and transcriptomic data.

Specific Google Cloud native services and supported tools are utilized in this module to automate and power bioinformatics data processing following the general workflow shown in Fig. 1, which illustrates the Google Cloud architecture used in the creation of the module as seen from the perspectives of both the developer and the user. The figure demonstrates the essential elements used in the creation of modules, such as Google Storage Buckets, Nextflow, and R Jupyter Notebooks, and elucidates that users have the option to utilize pre-existing modules to acquire knowledge about the integration of epigenetic and transcriptomic data. The cloud storage buckets described allow object storage to be utilized to store genomic data, such as raw sequencing information in FASTA format or aligned sequences in BAM format. The training module also utilizes Google Batch, a fully managed service that lets you schedule, queue, and execute batch processing (the method computers use to periodically complete high-volume repetitive data jobs) workloads on Google Cloud resources. Google Batch allows for workflow orchestration in several workflow languages, including Nextflow powered by Compute Engine virtual machines. Nextflow maintains a collection of ready-to-use workflows in a repository called Nextflow core (nf-core) that must adhere to strict guidelines before being published; these guidelines are implemented to standardize workflows, ensure reproducibility, assure quality, and encourage community collaboration. Nextflow also allows cost labels to be added to Google Cloud profiles, letting users see the cost not only of the entire pipeline but of individual processing steps to inform users where optimizations may be needed. Because the focus of this module was epigenetic and transcriptomic data integration, MethylSeq and RNA-Seq nf-core pipelines were utilized to demonstrate the power of ready-made bioinformatics workflows running in the cloud environment.

Instructions to deploy this module and all the images used within it can be found in the README file located in the NIGMS Sandbox GitHub repository (https://github.com/NIGMS/Integrating-Multi-Omics-Datasets). For users with limited experience working with GC, tutorials for Google Cloud services categorized by their associated research methods can be accessed by navigating to the NIH Cloud Lab Google Cloud Platform Tutorial Resources page (https://github.com/STRIDES/NIHCloudLabGCP). The run time for all three modules varies and will mostly depend on the size of the data sets being processed. For example, if the provided GitHub modules run as-is with the mentioned dataset, submodule 1 takes ~10 min to install the libraries and dependencies and 2 min to download the dataset and perform the analysis. Submodule 2 takes ~6 min to install the libraries and 13 min to perform the analysis. For the integration submodule, the installation of the libraries takes about 45 min, and it takes another 30 min to perform the analysis and see the results. The machine type used for this analysis is N1-standard with 4 CPUs and 15 GB of RAM, with about 100 GB of disk space. Note that the data set used in this module is relatively small. Both the time and storage necessary for computation increase dramatically as modern methodologies create ever-growing amounts of data in an attempt to satiate the demand for quantitative biological information; in fact, it is estimated that the total amount of genomic sequencing data produced alone doubles every 7 months [43].

**Figure 2:**
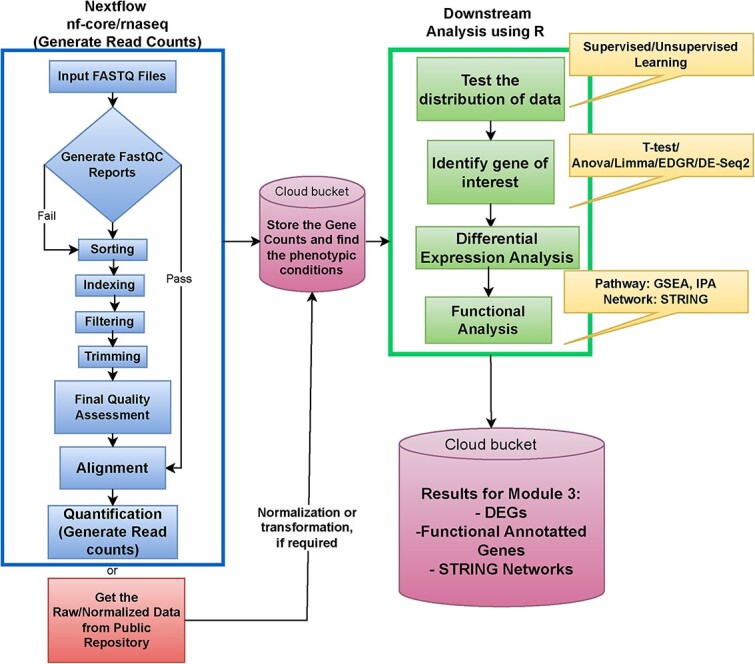
This figure demonstrates the analysis workflow of the RNA-Seq submodule. The blue section (box in the upper left-hand corner of the figure and under the title ``NextFlow nf-core/rnaseq'') generates read counts from raw data files (FASTQ) and explains the pre-processing steps in detail utilized through the Nextflow nf-core/rnaseq pipeline. The preprocessing steps will give you the read counts, or you can directly download the read counts from a public repository. The processed data will then be stored in the cloud bucket to perform the downstream analysis. The green section (upper right side under the title ``Downstream Analysis Using R'') shows a detailed description of the downstream analysis, where the results will be stored in the cloud bucket again to perform integration in submodule 3.

### Setting up the environment

To set up the environment, a Google Cloud account is first required. Vertex AI is Google Cloud’s AI platform, which includes tools like JupyterLab that are maintained by Google’s deep learning team. Initially, a user-managed R4.2 notebook within Vertex AI Workbench must be created with at least 8 CPUs and 30 GB of RAM. The n1-standard-8 virtual machine within Compute Engine meets these requirements. In addition, a service account with appropriate roles is required for Google Batch and must be created within IAM (identity and access management) or added to the default compute engine service account used in Vertex AI Workbench. If you are executing this tutorial with an NIH CloudLab account, your default Compute Engine service account will have all the required IAM roles to run the nextflow portion. Once within the notebook, the module can be cloned from GitHub using GitHub CLI (a tool designed to access the GitHub repositories through the command line environment) or through a menu option within Jupyter. To clone the repository, users can use the following command: git clone https://github.com/NIGMS/Integrating-Multi-Omics-Datasets.git. Additional modules can be found at https://github.com/NIGMS. The UND multi-omics repository contains three main folders representing each submodule, and every folder has its own Jupyter notebook, if applicable.

### Running the modules

The cloned repository has three folders for each submodule (RNA-Seq, RRBS, and Integration) and is designed to run on the R kernel. All the submodule folders consist of one .ipynb Jupyter notebook and the accompanying files required for the analysis. The initial cells of the notebook for the RNA-Seq and RRBS submodules contain data pre-processing commands that are executed through Nextflow. It is important to note that RNA-seq and bisulfite sequencing also have dedicated tutorials where the analysis is explained in more detail. For the RNA-Seq and bisulfite sequencing data, further preprocessing and normalization are performed using R packages. The list of all the necessary packages and their respective versions is provided under the ‘Software Requirements’ heading on the GitHub page for the module. Differential analysis, enrichment analysis, and visualization are performed in the same notebook. Further explanation of the code is provided in the markdown cells of the notebook. The Integration submodule uses the processed data results from the RNA-seq and RRBS submodules to incorporate both datasets into a wider inter-related analysis that provides a better understanding of the experiment.

## Module details

### Submodule 1: transcriptomics

Genomics data is often used as a starting point for a baseline analysis, and this is where submodule 1 begins. The intent of this submodule is to guide the user through the data processing protocols presented in Fig. 2. The first portion of submodule 1 leverages the power of the nf-core/RNA-seq pipeline, a genomics workflow created by the nextflow community, for transcriptomics data preprocessing [44]. This analysis pipeline is well established for finding biologically significant results in the integrated transcriptomics and multi-omics literature [45]. This data is then analyzed downstream using R, and the experimental results are returned to Google Cloud buckets for storage and visualization. The remainder of this section details this procedure step-by-step as illustrated in Fig. 2, beginning with data collection.

**Table 1:** An example of three sub-tables shown using first few rows and columns of different data files used for execution within these submodules. There is total three file samples: (1) demographic file, which provides the information about samples; (2) RNA sequencing normalized count file, which provides the information about the expression of gene; and (3) Bisulfite sequencing status of each CpG’s across each sample

GSE225846 Demographics sample					
ID	S_142_Gati_603_redo	S_53_Gati_506_redo	S_54_Gati_507_resent	S_72_Gati_526_redo	S_73_Gati_529_redo
GSM	GSM7058108	GSM7058109	GSM7058110	GSM7058111	GSM7058112
Samp_Type	Tumor	Normal	Tumor	Tumor	Tumor
Age	46	62	62	66	50
Ethnicity	African American	Caucasian	Caucasian	Caucasian	Caucasian
ER_Status	Positive	Positive	Positive	Positive	Positive
PR	Negative	Positive	Positive	Positive	Positive
HER2	Positive	Negative	Negative	Negative	Positive
Stage	IA	IIIC	IIIC	IA	IIB
**GSE225846 Processed Data Sample**					
	GSM7058144	GSM7058185	GSM7058209	GSM7058114	GSM7058127
ENSG003.14	6.9728	5.6314	8.0708	6.8707	6.0958
ENSG419.12	7.1300	7.5675	6.7341	7.3579	6.2121
ENSG457.14	7.6668	7.6124	8.1450	7.7911	8.1509
ENSG460.17	5.8652	6.1520	6.7439	6.3908	5.8803
ENSG938.13	5.7018	4.9637	7.8009	6.1936	5.9276
**GSE225847 processed data sample**					
	GSM7057513	GSM7057514	GSM7057515	GSM7057516	GSM7057517
AK097446	2.4603	2.9671	2.9896	2.4162	2.8780
AK097453	−4.9091	−4.9598	−4.5805	−4.7790	−4.6846
AK097470	−0.4811	−0.1591	−0.4303	−0.6537	−0.0845
AK097472	1.5207	1.6758	1.9436	1.8698	2.0732
AK097493	−3.1254	−2.8899	−3.0554	−2.7034	−1.9371
AK097500	0.7014	0.9981	1.3885	0.8310	1.2844

Public repositories, such as NCBI’s GEO, ArraryExpress, and The Cancer Genome Atlas (TCGA) are common platforms where researchers can find publicly available genomics data. GEO datasets can be located by their unique accession number. In this submodule, the user is analyzing GEO data series GSE225846, which is part of a super series and consists of gene expression data from breast cancer samples. The source of this data is an NCI-Maryland breast cancer cohort, including 185 tumor samples, 113 additional paired adjacent normal samples, and 104 normal tissues from reduction mammoplasty. For this submodule focusing only on gene expression, GSE225846 contains 155 samples consisting of 75 normal samples and 80 tumor samples ranging from stage IA to IIIC breast cancer in patients of varying ages and ethnicities. Provided in Table 1 is a sample of a few key files every user should be able to recognize, in order to become better acquainted with the data they are working with as well as the sample information; these include the demographics file and different series data files from the two GEO datasets used in this manuscript. Since the GEO data series did not have an annotation file accompanied with its respective platform, these submodules utilize the GPL16304 annotation file for probe identifiers and transcription start site (TSS) information, which becomes key in later submodules.

**Figure 3:**
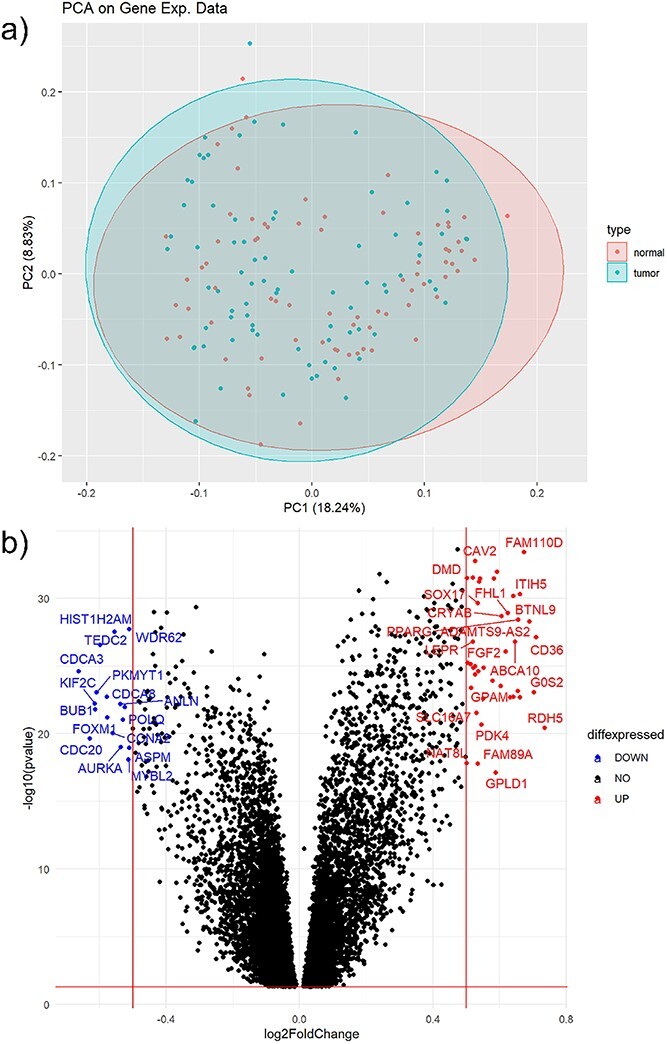
Gene expression intermittent results. (a) is a PCA to show the data distribution across the samples. (b) Volcano plot of significant gene (up and down regulated) with *P*-value and fold change between two phenotypic conditions.

The user has the option to begin the module using FASTQ files or raw sequencing data. If the user chooses to use the raw expression data, it will need to undergo normalization before proceeding to downstream analysis. If the user decides to use the FASTQ files, the data will be processed using nf-core/RNA-seq. A flow chart for this procedure can be found in the blue box in Fig. 2. The nf-core/RNA-seq pipeline begins with quality control checks on sequencing data using FastQC (a program designed to spot potential problems in high-throughput sequencing datasets) to help ensure the integrity of the data. Then read trimming is performed to remove any adapters or low-quality sequences so that only the highest fidelity reads are saved for downstream analysis. The trimmed reads are then aligned to the reference genome, as this tells the user which precise location in the genome each base pair in each sequencing read comes from. Post-alignment, the number of reads associated with each gene is counted to collect a quantitative view of expression, and nf-core/RNA-seq incorporates quality control metrics to help guarantee that the data sent to be analyzed downstream is of the highest utility.

As a confirmation to proceed with analysis, the user can look for density plots with a smooth curve and relatively shared intensity boxplot values in this dataset’s respective plots, as shown in Supplementary Fig. S1. Figure 3 shows the results of the gene expression analysis concurrent with the green boxes of Fig. 2 as the user makes their way through the module. Figure 3a shows the outcome of principal component analysis (PCA), which is a common unsupervised learning tool usually used for data visualization or pre-processing before supervised techniques are applied [46]. The plotted PCA results shown in Fig. 3a delineate the data distribution across tumor samples and normal samples. In the interpretation of PCA plots such as this, overlapping across samples would imply no technical bias and no inherent differences between populations. Conversely, non-overlapping regions would implicate an inherent difference between populations, which could be beneficial in the search for prognostic, diagnostic, and predictive genetic biomarkers. Note that bioinformaticians should apply PCA as one step out of many in their research flow to inform their analysis, as we did here, and not rely on it solely as a biological prognosticator. Hierarchical clustering is a supervised learning technique that is also included as an example in the downstream analysis workflow, in which genes with similar expression patterns are grouped together and connected by a series of branches as a cluster tree or dendrogram. Figure 3b shows the volcano plot of differentially expressed genes between the two phenotypic conditions via *P*-value and log2 fold change (log2FC) when comparing tumor versus normal tissue samples, the direct result of implementing the differential expression analysis from the green box in Fig. 2. For this example, while there were 9072 genes considered significant with regards to a *P*-value <0.05, no genes had a significant fold change of 2, which is found to be typical of gene expression data. Some quality control mechanisms available at this stage include checking for variability by comparing means, standard deviations, and variances found in the data. After conducting any functional analysis, such as gene set enrichment analysis (GSEA) and protein network investigations via STRING, the user will have the experimental results from submodule 1 in the form of differentially expressed genes, functionally annotated genes, and STRING protein networks returned to Google cloud buckets for later integration in submodule 3.

### Submodule 2: epigenomics

**Figure 4:**
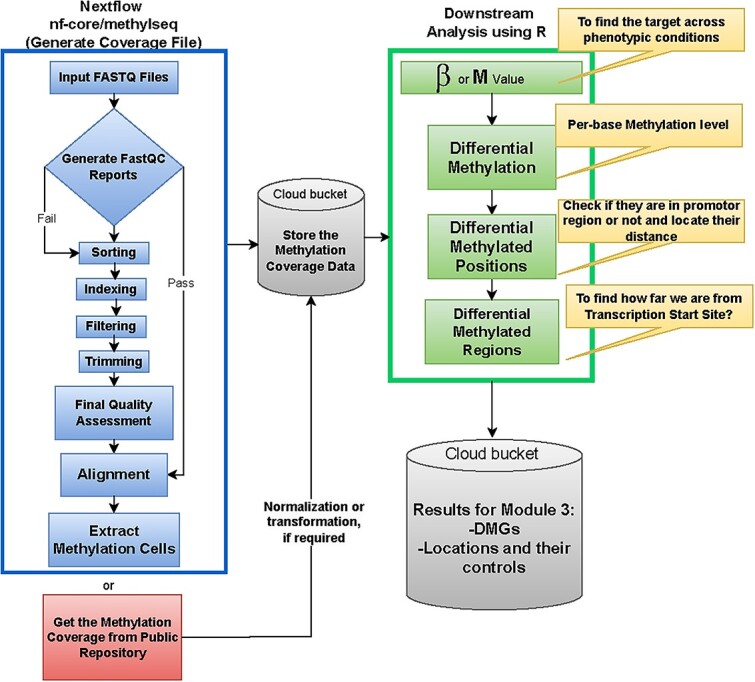
Figure shows the analytical workflow of the DNA methylation submodule. The blue section (box in the upper left corner of the figure and under the title ``NextFlow nf-core/methylseq'') generates the coverage file and explains the pre-processing steps in detail utilized through the Nextflow nf-core/methylseq pipeline. The preprocessing steps will provide the information on methylation cells, or researchers can directly download the methylation coverage files from a public repository. The processed data will then be stored in the cloud bucket to perform the downstream analysis. The green section (upper right side of the figure and under the title ``Downstream Analysis using R'') shows a detailed description of the downstream analysis, where the results will be stored in the cloud bucket again to perform integration in submodule 3.

This submodule uses the GEO data series (GSE225847), which is part of the super series with GSE225846 from submodule 1, and after processing and filtering, contained 595 samples, including 231 normal, 140 adjacent normal samples, and 224 tumor samples from patients of varying age, ethnicity, and sex. The intent of submodule 2 is to focus on the analysis of DNA methylation data by walking the user through the epigenomics workflow detailed in Fig. 4. The steps in the epigenomics data preprocessing pipeline, located in the blue box in Fig. 4, are very similar to the stages of the transcriptomics workflow described in submodule 1. Analogously, the user has the option to either conduct the pre-processing procedure with FASTQ files using nf-core/methylseq, a bioinformatics analysis pipeline used for methylation (bisulfite) sequencing data, or immediately get the raw methylation cells from a publicly available repository. Since the nf-core/methylseq pipeline is almost identical to the nf-core/RNA-seq workflow described in the previous section, we will proceed to describe the downstream analysis. When the effects on differentially methylated CpG sites are assessed, two types of outcomes can be used for statistical analysis: beta-values and M-values [47], and, i.e. what we will start with. The purpose is to observe the differential methylation states of the gene(s) to discover if they are switching between unmethylated and methylated states or vice versa within a particular phenotypic condition. The location, distance, and the differentially methylated genes will be stored in the Google Cloud buckets for integration in submodule 3. Submodule 2 provides data in the form of 2-channel, IDAT files to be processed within R. The data format is convenient for reading the methylation array, preprocessing the raw set, converting, and aligning to the reference genome where necessary, normalization, and filtering to a usable dataset for downstream analysis as beta- or M-values. Beta- and M-values can be visualized with histograms and MA plots, respectively. Typically, histograms representing methylation percentage have peaks at both extremes of the range. While not rigidly defined or followed, regions of percentage may be categorized into ‘unmethylated’ being methylation levels ranging from 0% to 10%, while ‘fully methylated’ may be samples between 90% and 100% [48, 49]. Based on the results, patterns may arise across several cells in terms of high and low methylation. Similarly, the MA plot visualizes the differences between two populations (i.e., tumor versus normal) by transforming the data onto M (log ratio) and A (mean intensity) scales.

**Figure 5:**
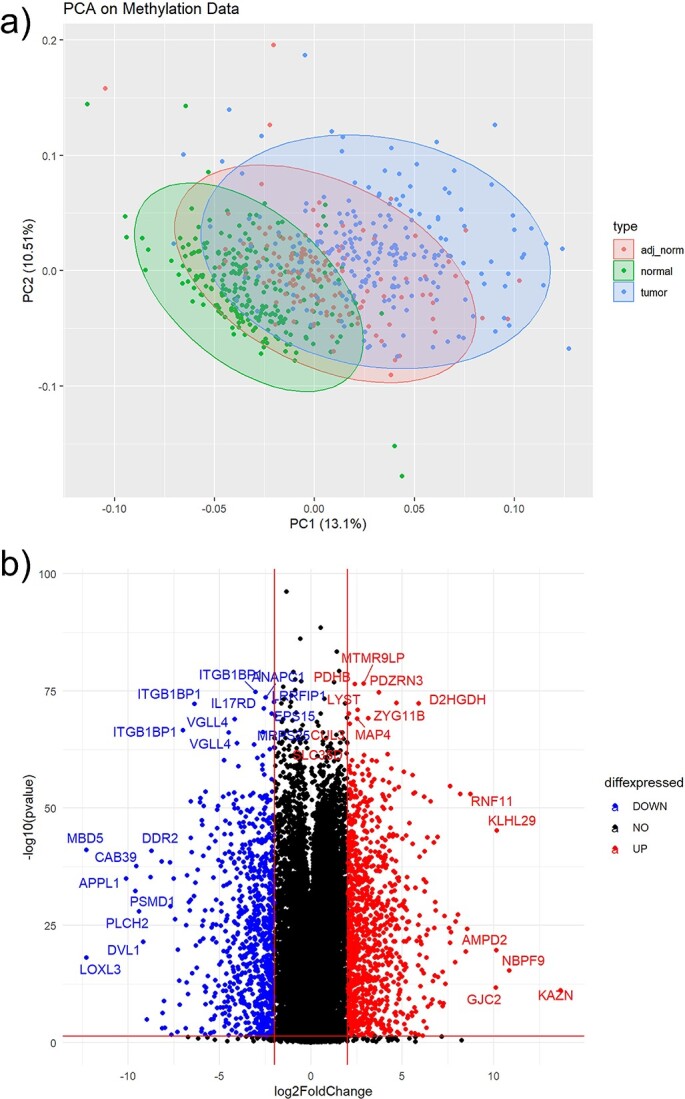
Methylation data intermittent results. (a) is a PCA to show the data distribution across the methylation profiles. (b) Volcano plot of significant CpGs (up and down regulated) with *P*-value and fold change between two phenotypic conditions.

Continuing with our downstream analysis, we move from the differential methylation step to the differential methylated positions and regions stages in our green box in Fig. 4. Understanding where methylation occurs gives insight into how methylation influences expression. These steps involve looking at whether or not the methylated site lies within the promoter region of the gene or some other area of influence, and checking to see how far the differentially methylated region lies from TSS. Expected results for this submodule are shown in Supplementary Fig. S2 using histograms and MA plots to visualize the data distribution. To maintain continuity with submodule 1, the PCA and volcano plot for submodule 2 are also presented here in Fig. 5a and b, respectively. Figure 5a seems to show a greater separation between phenotypic conditions than our previous PCA plots, indicating a possible genetic difference in the population. Figure 5b shows a subset of the statistically significant methylation probes as an example visualization for the user. It is typical with methylation data to observe a very large number of probes flagged as significant with regards to *P*-value < 0.05 and log2 fold change magnitude of 2 compared to the equivalent genomic data results. As expected, though, multiple probes may be in different regions of influence to the same gene’s TSS and reduce what would be considered a unique list of genes. When conducting regression analysis, variables, such as age, sex, smoking status, and other relevant factors are needed. It is important to acknowledge that methylation has a role in modifying the expression of genes throughout the process of cell differentiation, resulting in heritable modification [50]. As an example, individuals who currently engage in persistent smoking may have specific genes that are either hypomethylated or upregulated when compared with those individuals who have never smoked or have quit smoking [51, 52]. Using the genomation package [53] for the identification of differentially methylated regions and bases may provide valuable assistance in the biological interpretation of the acquired data.

### Submodule 3: integration

Integrating epigenomic and transcriptomic data has emerged as a pivotal strategy in bioinformatics to unravel the regulatory mechanisms underlying gene expression and cellular function [54, 55]. Existing integration techniques involve correlating DNA methylation patterns, histone modifications, and non-coding RNA with gene expression levels, enabling comprehensive insights into epigenetic regulation [56]. Advanced computational tools, such as chromatin conformation capture and co-expression network analysis, facilitate the integration of these multidimensional datasets, enabling the identification of regulatory elements and pathways [57].

**Figure 6:**
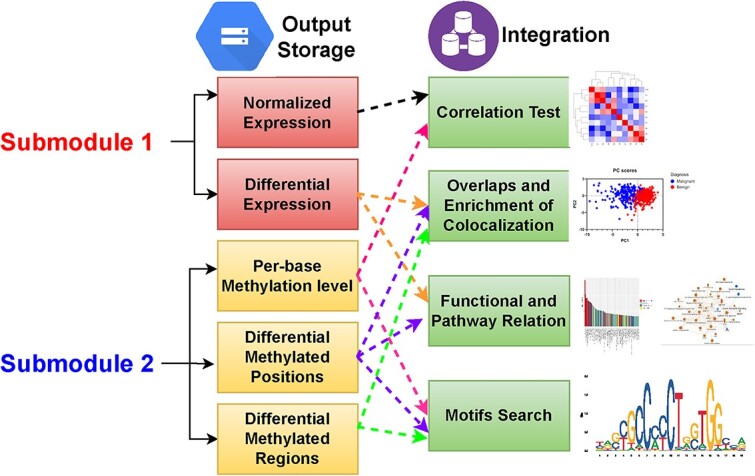
The figure represents the integration of information obtained from submodule 1 (RNA seq) and submodule 2 (Bisulfite). This is a flow of analytical information demonstrates the different steps which provides a collective information about the behavior of genes in terms of correlation, functional, pathways, and motif when examine expression and DNA methytion together.

**Figure 7:**
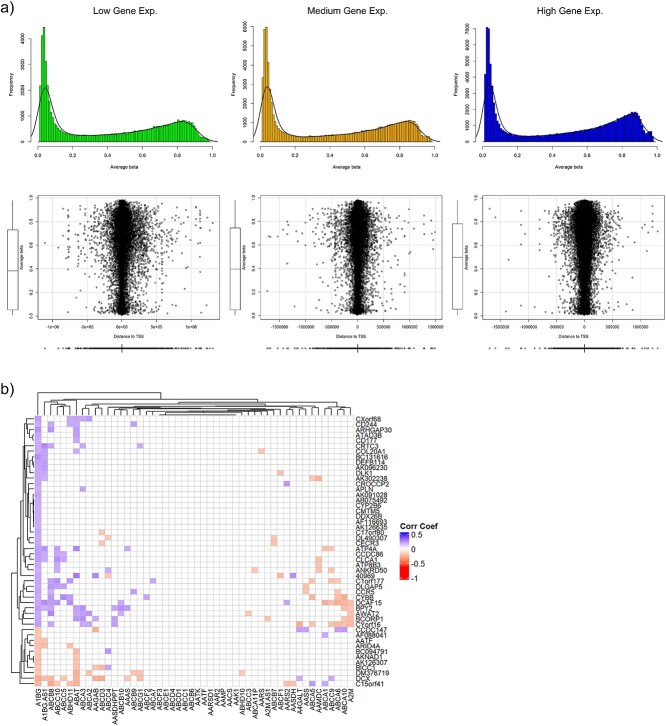
Association between DNA methylation and gene expression. (a) Mean methylation levels plotted according to three tertiles (low, medium, and high) of gene expression levels. Underneath a scatter plot of mean methylation levels in each gene expression tertile as a function of the distance from the transcription start site. The distribution of average methylation values and data across the CpGs with respect to distance from transcription start site are visualized by boxplots. (b) A heatmap of correlation matrix where one might see changes in methylation being positively- or negatively correlated with changes in another’s gene expression.

In submodule 3, the experimental results from the previous two submodules are integrated through various overlapping methods of analysis, as shown in Fig. 6, to try to identify patterns in the shared information between the genomic and epigenomic platforms. A large portion of this submodule is devoted to observing changes in methylation levels (beta values) with respect to transcription start sites (TSS distance) and changes in gene expression profiles. As depicted in Fig. 7, gene expression is split into three percentile groupings reflecting low, medium, and high expression. The corresponding beta-values for those genes are shown in the histograms at the top of Fig. 7a, while the distance between the methylated sites and the closest TSS is shown on the scatterplot below (bottom of Fig. 7a). These plots are also in agreement with established literature on the breast cancer epigenome, which likewise denotes an inverse relationship between lower methylation and high gene expression compared to the other levels (noting the higher frequency of lower beta-values) [42]. Methylation probes of interest to researchers may lie within the promoter region of a gene or roughly within 500 base-pairs of the gene’s TSS. This allows for various interpretations when looking at TSS distance scatterplots since, e.g. the high gene expression group in Fig. 7a has a higher density of methylation probes toward 0 compared to the other scatterplots.

Another overlapping integration tool for finding potential control mechanisms while accounting for a complex network of biological influences is analyzing the correlation between methylation data and gene expression. For these datasets, over 12 000 genes were significantly expressed (*P*-value < 0.05) and over 35 000 significant methylation probes were identified, with ~10 000 genes overlapping between these two lists, yet by looking at where the methylated fold change overlaps with statistically significant changes in gene expression, that list of interest shrinks to a more manageable number of about 1000 genes. The Pearson correlation coefficient was calculated for all 10 000 common genes (for proof of concept) between the differential methylation data and the gene expression data. Because the sheer size of the resulting matrix is too large for depiction here, Fig. 7b shows a sample 50 × 50 subset with genes from the gene expression dataset along the *x*-axis and genes from the methylation dataset along the *y*-axis. Notice that the expression data of the first gene (A1BG) has both positive and negative correlation with the 50 shown differentially methylated genes along the *y*-axis, as is the case for many other genes from the expression data set throughout the entire correlation matrix. This is a prime example of the integration of genomic and epigenomic data presented through the analytical lens of a single statistical mechanism that allows for a deeper biological understanding than would have been possible otherwise.

**Figure 8:**
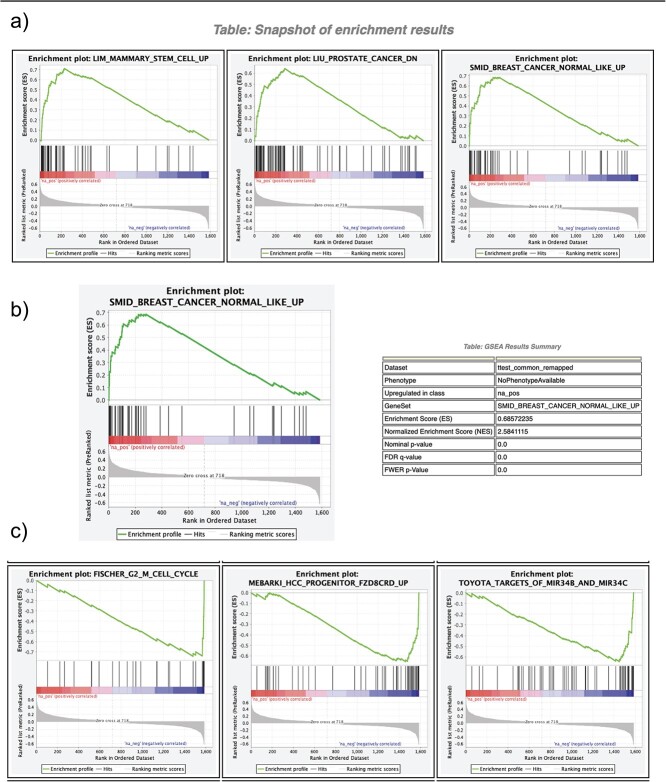
Multi-omics integration functional analysis. This figure is the output of gene set enrichment analysis (GSEA), which is an open-source software used for functional analysis of pre-ranked gene lists. The gene list used for this is the overlapping significant genes from both gene expression and methylation datasets with regards to the *P*-value and log2 fold change when comparing normal to tumor samples. (a) shows statistically significant up-regulated pathways while (b) is a detailed breakout of a statistically significant up-regulated pathway that is relevant to breast cancer subtypes, which is relevant to our findings and previously established processes, and (c) shows statistically significant down-regulated pathways.

We finish module 3 by briefly looking at the functional relevance of our findings: 1588 statistically significant genes from the multi-omics integration were analyzed using GSEA, a computational method that determines whether an a priori defined set of genes shows statistically significant, concordant differences between two biological states [58, 59]. Again, for concise visualization, a few plots are shown in Fig. 8 for the reader to digest. The enrichment score is simply the value with the largest magnitude from the given enrichment plot. Genes before this point are the subset members contributing the most to this score and are most interesting if appearing early in the given pathway since the input gene list is ranked based on *P*-value, fold-change, or another user-defined metric. Figure 8a and c show pathways flagged as statistically significant given their positive (up-regulated) and negative (down-regulated) enrichment scores, respectively. Figure 8b presents a single pathway with additional information in a sample GSEA results summary table, highlighting the enrichment score from the corresponding plot and statistical parameters of significance while also showing a calculated normalized enrichment score, which can be used to compare analysis results across gene sets. A pathway related to breast cancer subtypes is emphasized here, giving further confirmation to previous results found by Singhal *et al.*, as this collection of genes is up-regulated in the normal-like subtypes of breast cancer. The integration of datasets from different multi-omics fields is becoming essential for developing effective and comprehensive bioinformatics analysis pipelines for scientific and clinical research. This training module establishes a protocol for integrating transcriptomic and epigenetic data utilizing the cloud computing infrastructure on Google Cloud, with an emphasis on practical application geared towards informing and aiding the workflow of biological researchers.

## Discussion

Multi-omics research has become increasingly important for the analysis of complex biological pathologies. The integration of genomics and epigenomics is increasingly recognized as crucial in understanding the complexities of biological systems and their implications for health, disease, and evolution. Many diseases, including cancer, neurological disorders, and metabolic syndromes, involve both genetic and epigenetic alterations. Integrative genomics and epigenomics studies can uncover the interplay between genetic mutations and epigenetic modifications, leading to the identification of disease mechanisms and potential biomarkers for diagnosis, prognosis, and treatment.

The primary objective of this training module is to demonstrate the process of acquiring, preparing, analyzing, and integrating transcriptomic and epigenomic data to produce useful scientific results for the bioinformatics researcher, with the focus being on the integration of the two data types. After completing this module, the user will possess a greater understanding of how to integrate epigenetic DNA methylation data and transcriptomic RNA sequencing data analysis into their workflow to solve complex biological problems. As more DNA methylation data has become increasingly available, a complicated and dynamic relationship between DNA methylation and gene expression is coming into view. By combining the DNA methylation data in a biological system with the transcriptomic information, the investigator can go beyond the simple analysis of how genes are being expressed to probe the regulation and control of such expression, and to link epigenetic patterns with specific phenotypic conditions in cells. This combination of genomics, which is concerned with the structure, function, and mapping of the genome, with epigenomics, which investigates the epigenetic changes in the genetic material in the cell, is vital to forming a complete understanding of phenotypic expression.

To prove the scientific relevance of our module, we are providing a summary of previously published papers wherein we evaluated gene expression data and DNA methylation data in the analysis of breast tissues. Breast cancer in patients can be molecularly characterized by parameters, such as ER or HER2 status, and generally fall into four subtypes determined by gene expression patterns: Luminal A, Luminal B, HER2+, and Basal (triple-negative breast cancer). Three genes (ESR1, ERBB2, and AURKA) or a set of genes have been used to classify the BC patients [60]. By incorporating data sets consisting of high-throughput DNA methylation profiling in human frozen breast tissue, our lab was able to extract some useful biological information. Performing a hierarchical clustering analysis on the DNA methylation profiling data identified two main breast cancer categories stratified by ER status, similar to gene expression biomarkers, with one cluster showing predominantly ER+ methylation and the other displaying predominantly ER-methylation. In addition, unsupervised analysis of recurrent methylation patterns yielded six distinct groups of tumors, termed clusters 1–6, displaying differences in expression subtype composition and clinical characteristics beyond the classic four molecular subtypes. This importantly demonstrates the complexity and heterogeneity of breast cancer and the potential of DNA methylation profiling to refine breast tumor taxonomy. Our work also compared the gene expression signatures of several normal mammary epithelial subpopulations [61] with the gene expression and DNA methylation profiles of our six DNA methylation-based groups of patients in the main data set. These observations suggest that the methylation patterns we have identified might be related to the cell type of origin of the tumors concerned. This newly discovered scientific information is of interest to both breast cancer researchers who want to understand more about the heterogeneity of breast cancer and clinicians who want to be able to deliver more accurate diagnoses, prognoses, and provide therapeutical approaches that are more targeted to the patient from a genetic standpoint. By investigating the epigenomic and genomic environments for phenotypically heterogeneous tumors, we were able to extract new information from the data that is both biologically novel and clinically meaningful, information that could aid in breast cancer detection and classification. Utilizing the approaches presented in these three submodules will provide the module user with the necessary skills, knowledge, and experience to likewise extract scientifically and clinically relevant information from large, complex epigenetics and transcriptomic data sets and contribute to the wider body of scientific knowledge.

To bolster this effort, the training module is designed to be intuitive, accessible, and low cost for new bioinformaticians and novice users, leveraging the computational resources, scalability, and affordability of Google Cloud to efficiently store, manage, and analyze the large multi-omics data sets involved. One of the most instrumental components of the training module is its reliance on cloud computing infrastructure. By allowing researchers remote access to many servers that can process information simultaneously, cloud computing facilitates the analysis of enormous quantities of data with great efficiency, avoiding costly hardware purchases and costs associated with physical server space. This is particularly convenient with respect to multi-omics research, which often entails processing massive amounts of biological data. Google Cloud has provided a platform for the data storage and computational resources necessary to run this training module and has made it possible for users from all over the world to access its contents at relatively low cost. The training module we have outlined in this paper costs the user ~$3.20 to run end-to-end, assuming everything is shut down and all resources are deleted upon completion. These three submodules are designed to guide the user through the workflow of integrating multi-omics data, especially epigenomic and genomic data, into their analysis and provide the knowledge, tools, and skillset that will be necessary for the next generation of bioinformatics researchers to tackle complex biological problems. This training module is useful for bioinformaticians with all levels of experience who want to expand their skill set and improve their understanding of genomic and epigenomic data analysis.

## Conclusion

The module’s design stands out for its unique and highly useful approach to addressing the challenges posed by the burgeoning field of multi-omics research. In the future, this kind of comprehensive approach will allow researchers to investigate DNA, RNA, proteins, metabolites, epigenetic changes, and environmental exposures, creating a holistic view of complex disease pathologies. What sets this module apart is its focus on empowering investigators with limited experience in multi-omics methodologies. The cloud-based training module, hosted on GC, guides users through the integration of transcriptomic and epigenetic data, which is particularly vital in cancer research. Given the vast amounts of data generated by omics research, efficient computational platforms like GC are essential for managing and analyzing this wealth of information. The scalability, flexibility, and collaborative features of GC make it an ideal tool for bioinformatics research.

In conclusion, integrating the analysis of genomics and epigenomics is crucial for advancing our understanding of biology, disease, and evolution. It enables researchers to unravel complex regulatory networks, discover biomarkers, develop personalized medicine approaches, and address pressing health and environmental challenges.

**Key Points**

- The integration of transcriptomics and epigenomics data analysis is becoming an increasingly important tool for understanding complex biological disease states, identifying new prognostic, diagnostic, or predictive biomarkers, uncovering novel therapeutic targets, and developing more personalized treatment protocols for patients.
- Our team has designed a cloud-based learning module on Google Cloud to teach investigators who have limited familiarity with multi-omics approaches how to incorporate transcriptomics and epigenetics data analysis and integration protocols into a comprehensive analysis pipeline for their own work.
- The instructional analysis pipeline presented here consists of three submodules: a transcriptomics module using RNA-seq data, an epigenomics module employing RRBS (reduced-representation bisulfite sequencing) data, and an integration module. The content covered in the module tutorials is presented in the form of breast cancer case studies and includes data retrieval, data processing, integration, and downstream analysis.

## Supplementary Material

SuppFig1_bbae352

SuppFig2_bbae352

## Data Availability

The data that supports the findings of this study are openly available in Gene Expression Omnibus (GEO) with the reference identifiers as GSE225846 at https://www.ncbi.nlm.nih.gov/geo/query/acc.cgi?acc=GSE225846 GSE225847 at https://www.ncbi.nlm.nih.gov/geo/query/acc.cgi?acc=GSE225847. All module material and code associated with this submission is available on GitHub at https://github.com/NIGMS/Integrating-Multi-Omics-Datasets.
