# Supplementary figures and images for: Transcriptomics and epigenetic data integration learning module on Google Cloud
Source: Brief Bioinform. 2024 Aug 5;25(Suppl 1):bbae352. doi: 10.1093/bib/bbae352 (PMC11299028; doi:10.1093/bib/bbae352)

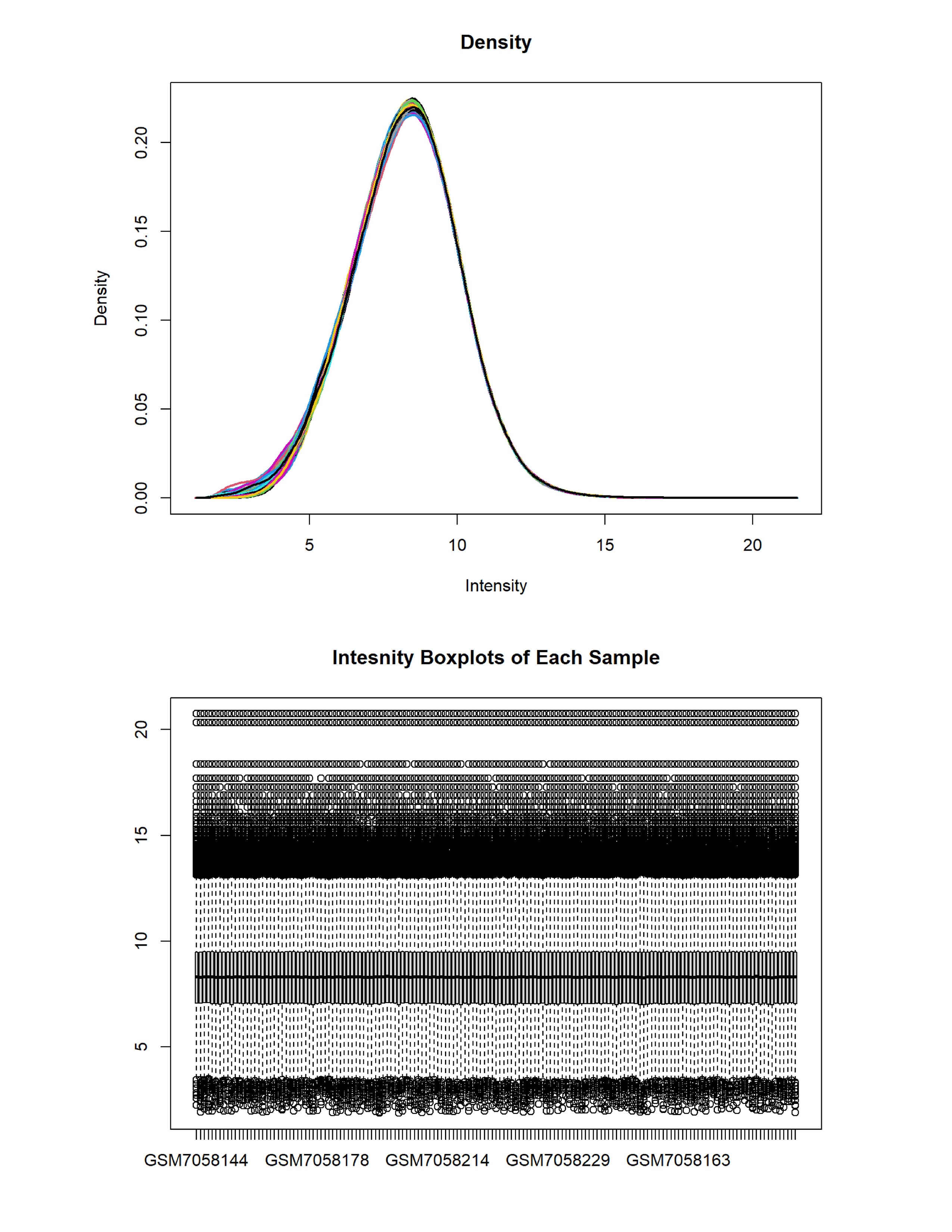

Supplement: SuppFig1_bbae352 [file suppfig1_bbae352.jpeg]

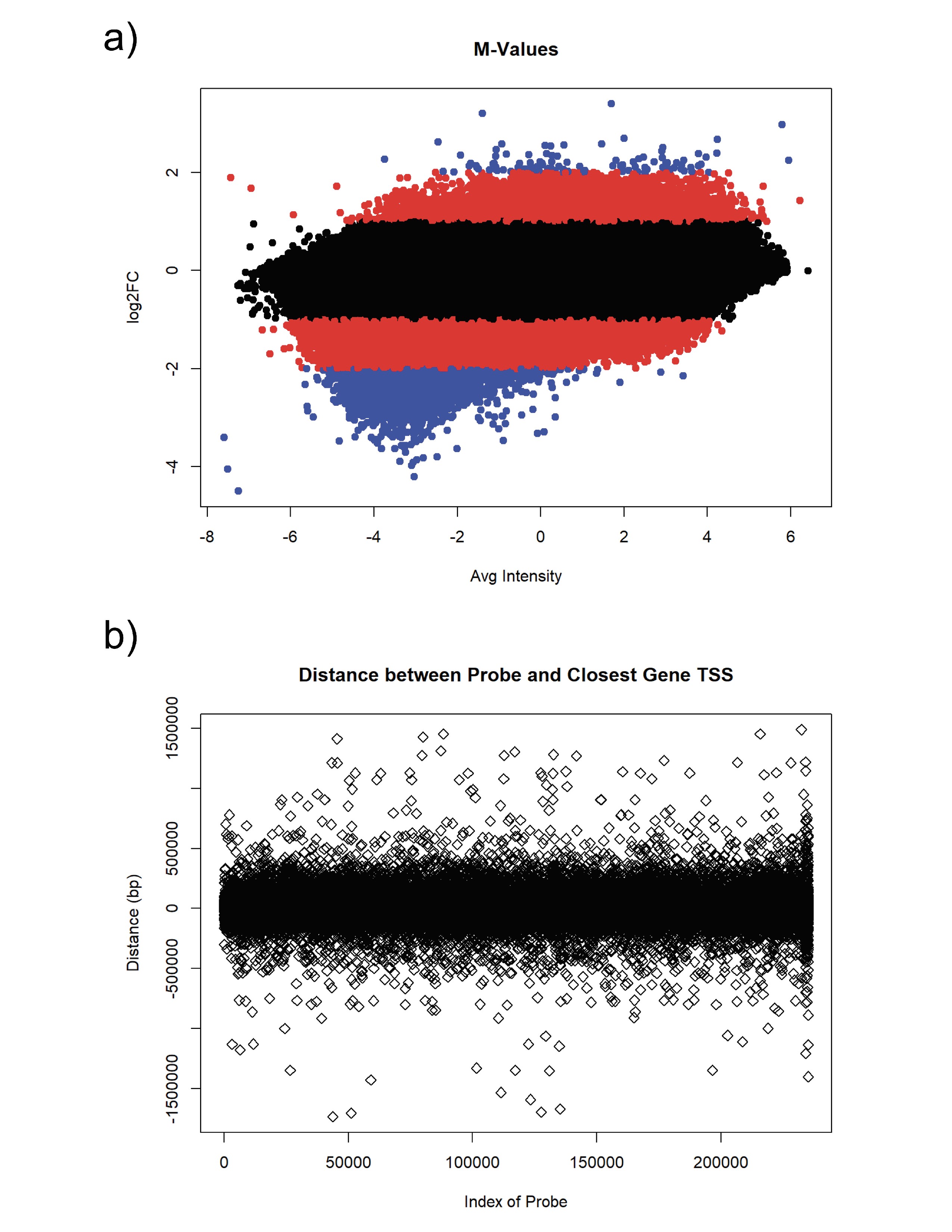

Supplement: SuppFig2_bbae352 [file suppfig2_bbae352.jpeg]
